# Two new species of the gorgonian inhabiting barnacle, *Conopea* (Crustacea, Cirripedia, Thoracica), from the Gulf of Guinea

**DOI:** 10.3897/zookeys.270.3736

**Published:** 2013-02-18

**Authors:** Dana Carrison-Stone, Robert Van Syoc, Gary Williams, W. Brian Simison

**Affiliations:** 1 Department of Invertebrate Zoology and Geology, California Academy of Sciences, 55 Music Concourse Dr., San Francisco, CA 94118, USA; 2 Center for Comparative Genomics, California Academy of Sciences, 55 Music Concourse Dr., San Francisco, CA 94118, USA

**Keywords:** Barnacle, Cirripedia, *Conopea calceola*, *Conopea fidelis* sp. n., *Conopea galeata*, *Conopea saotomensis* sp. n., COI, endemic, gorgonian, Gulf of Guinea, H3, host specificity, octocoral, phylogeny

## Abstract

Two new species of *Conopea* (Say 1822) are described from the Gulf of Guinea: *Conopea saotomensis* **sp. n.**and *Conopea fidelis* **sp. n.** These two new species were collected from the historically isolated volcanic islands of São Tomé and Príncipe. The relationship between *Conopea saotomensis* sp. n., *Conopea fidelis* sp. n.and two other Atlantic barnacle species, *Conopea calceola* (Ellis 1758) and *Conopea galeata* (Linnaeus 1771), is examined. The methods employed are the construction of a molecular phylogeny using mitochondrial COI and nuclear H3 gene sequence data along with morphological comparisons of calcareous and cuticular body parts. It is found that *Conopea saotomensis* **sp. n.**, *Conopea fidelis* **sp. n.**and *Conopea calceola* are most closely related to each other but the relationship among them is unresolved. Gorgonian hosts are identified. Preliminary observations show species level host specificity for *Conopea fidelis* **sp. n.**

## Introduction

The Gulf of Guinea island chain consists of Bioko, São Tomé, Príncipe, and Annobón. This study focuses on São Tomé and Príncipe, which are approximately 140 km apart and 274 km west of northern Gabon. They are the products of large shield volcanoes originating 3,000 m below the ocean’s surface along the Cameroon line. São Tomé and Príncipe are old islands, 13 and 30 myo, respectively, and have never been connected to the African mainland.

## Genus *Conopea*

Say (1822) designated a new genus *Conopea* to accommodate a new species, *Conopea elongata*, he described from eastern Florida. He included a previously known barnacle *Balanus galeatus* (=*Lepas galeata* Linnaeus 1771) in *Conopea*. *Conopea elongata* is later listed, by Darwin (1854), as a junior synonym of *Conopea galeata*. Say describes *Conopea* as ‘Shell sessile, fixed, composed of two cones joined by their bases, the lines of junction carinate each side: inferior cone entire, attached by its anterior side and tip to marine bodies; with an aperture at the summit, closed by a quadrivalved operculum.’

*Conopea* is a widespread genus that is found in temperate and tropical oceans around the world. Currently, there are 21 described species of *Conopea*. In general, *Conopea* is not a well documented group. There is very little data on host associations, species ranges are not well defined, published descriptions are often incomplete and occasionally contain questionable information. Darwin (1854) thought *Conopea* to be closely related to the genera *Megabalanus* and *Acasta* whereas Hoek (1913) thought *Conopea* to be closely related to *Balanus*. All species of *Conopea* live in an obligate commensal symbiotic relationship with either a gorgonian or an antipatharian. The barnacle lives almost completely covered by host tissue, the basis of its shell clasps the axis of the host, with only the opercular opening exposed.

### Atlantic species of *Conopea*

There are three known species of *Conopea* found in the Atlantic Ocean and Caribbean Sea: *Conopea calceola*, *Conopea galeata*, and *Conopea merrilli*. *Conopea calceola* was originally described from the Strait of Gibraltar, by Ellis (1758). *Conopea calceola* has subsequently been recorded from the Mediterranean to South Africa, the Persian Gulf to western Australia, the Indian Ocean, and Japan (see Newman and Ross 1976 for literature summary). *Conopea merrilli* was described from South Carolina (Zullo 1966) and has since been recorded from the west coast of Florida and Puerto Rico (see Newman and Ross 1976). *Conopea galeata* was described by Linnaeus in 1771 but no type locality was given and the type specimen is lost (Pilsbry 1916). Darwin (1854) gave a description of *Conopea galeata* (as *Balanus galeatus*) and listed its known localities as South Carolina, Florida, West Indies, and Central America. Pilsbry (1916) also gave a description of *Conopea galeata* (as *Balanus galeatus*) and listed the distribution as South Carolina to the West Indies and Central America, and southern California. The current distribution range of *Conopea galeata* is North Carolina through the West Indies, the Gulf of Mexico to Venezuela, southern California to Panama, and the Galápagos Islands (see Newman and Ross 1976).

Morphologically *Conopea merrilli* and *Conopea galeata* are clearly different and easily distinguishable from *Conopea calceola*, *Conopea saotomensis* sp. n., and *Conopea fidelis* sp. n.. *Conopea calceola* is morphologically similar to the Gulf of Guinea species and is therefore compared in detail to aid in future identifications. *Conopea galeata* was chosen over *Conopea merrilli* as an outgroup for molecular analysis because of its larger distribution range and greater availability of specimens.

### Materials and methods

Approximately 40 individuals of *Conopea saotomensis* sp. n. and 20 individuals of *Conopea fidelis* sp. n. were collected from São Tomé and Príncipe by Carrison-Stone, Van Syoc, and Williams in 2006 and 2009. Barnacles were collected from three different localities on São Tomé: Diogo Vaz (0°18.89'N, 6°29.39"E), Ponta Baleia (0°2.13'N, 6°33.51'E), and Ilheu Santana (0°16'N, 6°45.48'E) and two different localities on Príncipe: Ilheu BomBom (1°42'8.8"N, 7°24'14"E) and Pedra de Galé (1°43'30.1"N, 7°22'51.5"E). Collections were done via SCUBA at depths of 9–33m. Seven individuals of *Conopea calceola* were collected from 3 separate sites at Porto Covo, Portugal, by Van Syoc in 2008. Samples of the associated gorgonian were also collected. All specimens were preserved in 95% EtOH.

*Conopea galeata* from St. Catherine Is., Georgia (USA) were borrowed from the California Academy of Sciences Invertebrate Zoology Department (CASIZ). *Conopea galeata* from South Padre Is., Texas and Mexico Beach, Florida were collected by Mary Wicksten. *Conopea galeata* from Port Aransas, Texas were collected by Carol Cox.

Barnacle cirri, mouthparts and opercular plates from São Tomé, Príncipe, and Portugal specimens were dissected for morphological comparisons. These physical traits, along with shell shape, in particular basis shape and presence/absence of longitudinal tubes in shell wall plates, are traditionally used for identification. The cirri and mouthparts were mounted on microslides and photographed at 100x with a Leitz microscope imaging system. Images of the opercular plates were taken with a scanning electron microscope (SEM, LEO/Zeiss 1450VP).

Identification of host gorgonians was based on external and sclerite morphology. Branching patterns, polyp shape, color and sclerite types were examined. Sclerites were isolated by dissolving small amounts of gorgonian tissue in sodium hypochlorite solution, followed by rinsing with water and then 75% ethanol. Images of the sclerites were taken with SEM and Leitz optical microscope imaging systems. All gorgonians harboring barnacles were identified using Grasshoff (1988, 1992). However some barnacle specimens lacked host tissue and were found attached to only the gorgonian axis. Therefore, identification of those hosts was impossible.

Genomic DNA was extracted from adductor muscle tissue using the Qiagen DNeasy Blood and Tissue kit (Valencia, CA). The cytochrome c oxidase subunit I (COI) primers COI-N: TGAGAAATTATTCCGAAGGCTGG (Van Syoc 1994, 1995) and LCO 1490: GGTCAACAAATCATAAAGATATTGG (Folmer et al. 1994) were used to amplify approximately 700 base pairs of the mitochondrial genome (mtDNA). Additionally, the Histone 3 primers H3F: ATGGCTCGTACCAAGCAGAC VGC and H3R: ATATCCTTRGGCATRATRGTGAC (Colgan et al. 1998) were used to amplify approximately 350 base pairs of the nuclear protein coding gene (nDNA). The COI thermal profile was an initial step of 94°C for 3 min, then 35 cycles of: 94°C for 30sec, 47°C for 30sec, and 72°C for 1min. H3 thermal profile was 3 initial steps of 94°C for 3 min, 50°C for 2 min, 72°C for 2 min, then 35 cycles of: 94°C for 35 sec, 50°C for 30 sec, and 72°C for 40 sec. The resulting sequence data were edited in Sequencher 4.7 (Gene Codes) and BioEdit 7.0.9 (Hall 1997). Alignments were initially performed with ClustalW 1.8 and then edited by hand.

Molecular phylogeny was determined by Bayesian and likelihood analyses. *Semibalanus balanoides* (GenBank accession AF242660.1), another archaeobalanid, was used as an outgroup. Bayesian analyses were run in Mr. Bayes (Huelsenbeck and Ronquist 2001) for 50 million generations with a sample frequency of 1000 using the CAS CCG PhyloCluster (a 280-core Apple Xserve High Performance Computing Cluster with 8–12 GB RAM/node (232 GB total)). The concatenated dataset was partitioned into 1^st^, 2^nd^, and 3^rd^ codon positions so that models of substitution could be estimated for each site with Mr.Modeltest 2.3 (Nylander 2004). Burn-in and convergence values were determined using Tracer v1.5 (Drummond and Rambaut 2007). Likelihood analyses were run in PAUP* 4.0b10 (Swofford 2003). Heuristic searches were performed along with bootstrap analyses; 10,000 bootstrap replicates with 10 random sequence additions to each bootstrap. The best-fit DNA substitution models were determined with Mr.Modeltest. All analyses were performed on the gene datasets separately as well as concatenated. Uncorrected nucleotide pairwise-distance matrices among and within groups were determined in MEGA 5.05 (Tamura et al. 2007).

**Table 1.** Data associated with the specimens used in this study.

Barnacle taxon	Gorgonian host	CASIZ Catalog	GenBank accession’s	Collection Locality
*Conopea calceola*	*Eunicella verrucosa*	175916	HQ290142, HQ290155	Porto Covo, Portugal
*Conopea calceola*	*Eunicella verrucosa*	175917	HQ290143, HQ290156	Porto Covo, Portugal
*Conopea calceola*	*Eunicella verrucosa*	180065	HQ290135, KC349910	Porto Covo, Portugal
*Conopea galeata*	unknown	106216	HQ290146, HQ290147	St.Catherine Is., Georgia
*Conopea galeata*	unknown	184331	JQ966287, JQ966283	South Padre Is., Texas
*Conopea galeata*	*Leptogorgia setacea*	183496	JQ966288, JQ966284	Port Aransas, Texas
Conopea galeata*	unknown	184416A 184416B	JQ966289, JQ966285, JQ966290, JQ966286	Mexico Beach, Florida
*Conopea saotomensis* sp. n.	*Leptogorgia viminalis*	173189	HQ290134, HQ290149	Diogo Vaz, São Tomé
*Conopea saotomensis* sp. n.	*Eunicella kochi*	173190	HQ290136, KC349911	Diogo Vaz, São Tomé
*Conopea saotomensis* sp. n.	*Leptogorgia ruberrima*	174321	KC349913, KC349922	Ilheu Santana, São Tomé
*Conopea saotomensis* sp. n.	*Leptogorgia dakarensis*	174804	KC349904, KC349916	Diogo Vaz, São Tomé
*Conopea saotomensis* sp. n.	*Leptogorgia varians*	174805	KC349906, KC349917	Diogo Vaz, São Tomé
*Conopea saotomensis* sp. n.	*Leptogorgia gaini*	174806	HQ290152, KC349918	Diogo Vaz, São Tomé
*Conopea saotomensis* sp. n.	*Leptogorgia ruberrima*	175525	KC349907, KC349919	Diogo Vaz, São Tomé
*Conopea saotomensis* sp. n.	*Leptogorgia dichotoma*	175526	KC349908, KC349920	Diogo Vaz, São Tomé
*Conopea saotomensis* sp. n.	unknown	178662	KC349909, KC349925	Diogo Vaz, São Tomé
*Conopea saotomensis* sp. n.	*Leptogorgia dakarensis*	178655	HQ290137, HQ290159	Bom Bom Is., Príncipe
*Conopea saotomensis* sp. n.	unknown	178656	HQ290160, KC349924	Bom Bom Is., Príncipe
*Conopea saotomensis* sp. n.	*Leptogorgia* sp.	180025	JQ966291	Pedra de Galé, Príncipe
*Conopea saotomensis* sp. n.	*Leptogorgia dichotoma*	185253		Diogo Vaz, São Tomé
*Conopea fidelis* sp. n.*	*Muriceopsis tuberculata*	174803A 174803B	HQ290140, HQ290151, KC349905, KC349915	Diogo Vaz, São Tomé
*Conopea fidelis* sp. n.	*Muriceopsis tuberculata*	174320	KC349912, KC349921	Ponta Baleia, São Tomé
*Conopea fidelis* sp. n.	*Muriceopsis tuberculata*	174322A 174322B	HQ290140, HQ290150, KC349914, KC349923	Ponta Baleia, São Tomé
*Conopea fidelis* sp. n.*	*Muriceopsis tuberculata*	178651A 178651B	HQ290138, HQ290139, HQ290157, HQ290158	Pedra de Galé, Príncipe
*Conopea fidelis* sp. n.	*Muriceopsis tuberculata*	185252		Ponta Baleia, São Tomé

* Two barnacles were used from this lot.

## Results

### Molecular analyses

**Figure 1.**
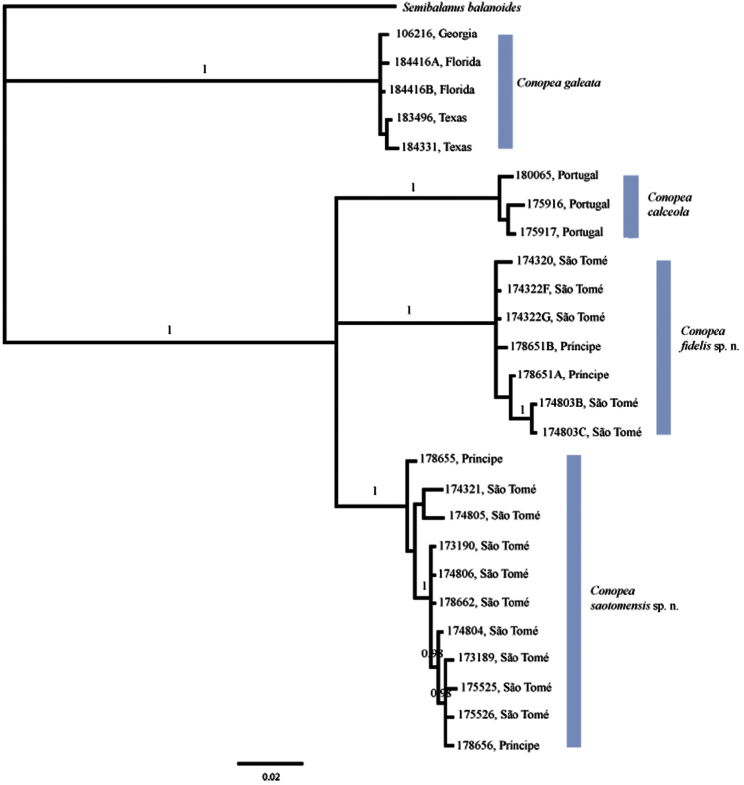
Bayesian phylogeny based on concatenated H3 nDNA and COI mtDNA sequences. Posterior probabilities of 0.95 or greater are shown. The relationship among the Gulf of Guinea species and *Conopea calceola* is unresolved.

Two major clades resulted from molecular analysis. One clade contains *Conopea calceola*, *Conopea saotomensis* sp. n.and *Conopea fidelis* sp. n, and the other contains *Conopea galeata*. Unfortunately, the gene data used was not sufficient to completely resolve the relationship among the three eastern Atlantic species. We do know that they are each other’s closest relatives but we do not know which two of the three are most closely related. Bayesian (Fig. 1) and likelihood phylogenies, based on concatenated COI and H3 datasets, between *Conopea saotomensis* sp. n., *Conopea fidelis* sp. n., and *Conopea calceola* are unresolved. Bayesian phylogeny generated solely on COI data shows the two Gulf of Guinea species as being more closely related to each other than to *Conopea calceola*. However the likelihood phylogeny generated with solely COI data again showed an unresolved relationship among *Conopea saotomensis* sp. n., *Conopea fidelis* sp. n., and *Conopea calceola*. Separate Bayesian and likelihood analyses of H3 sequence data showed similar unresolved topologies among the three eastern Atlantic species.

Pairwise uncorrected p-distances (Table 2) of COI and H3 also could not resolve the relationship. Distances for COI indicate that *Conopea saotomensis* sp. n.and *Conopea fidelis* sp. n. are more closely related to each other (8.2%) than to *Conopea calceola* (8.8% and 10.4%, respectively) whereas H3 distances indicate that *Conopea saotomensis* sp. n.and *Conopea fidelis* sp. n. are more closely related to *Conopea calceola* (1.4% and 1.3%, respectively) than to each other (2.2%). Pairwise uncorrected p-distances within groups is as follows: *Conopea saotomensis* sp. n. = 0.8%/0.0%, *Conopea fidelis* sp. n. = 0.7%/0.2%, *Conopea calceola* = 0.7%/0.0%, *Conopea galeata* = 0.3%/0.1% (COI/H3 respectively).

**Table 2.** Uncorrected pairwise distances among groups, COI (lower half of matrix) and H3 (upper half of matrix).

Barnacle taxon	*Conopea saotomensis* sp. n.	*Conopea fidelis* sp. n.	*Conopea calceola*	*Conopea galeata*
*Conopea saotomensis* sp. n.		0.022	0.014	0.110
*Conopea fidelis* sp. n.	0.082		0.013	0.106
*Conopea calceola*	0.088	0.104		0.102
*Conopea galeata*	0.148	0.165	0.166	

### Systematics

Subclass Cirripedia Burmeister, 1834

Superorder Thoracica Darwin, 1854

Order Sessilia Lamarck, 1818

Suborder Balanomorpha Pilsbry, 1916

Superfamily Balanoidea Leach, 1817

Family Archaeobalanidae Newman & Ross, 1976

Genus *Conopea* Say, 1822

#### Conopea saotomensis sp. n.

urn:lsid:zoobank.org:act:1164CCD8-C9F3-46E2-BBB9-1F6411123DA7

http://species-id.net/wiki/Conopea_saotomensis

**Figure 2.**
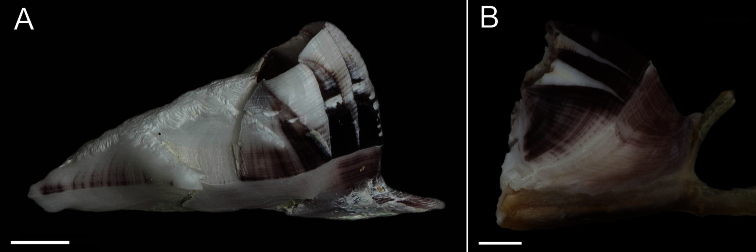
*Conopea saotomensis* sp. n., **A** whole shell (CASIZ174804) **B** whole shell attached to gorgonian axis (CASIZ174806). Scale bar = 2 mm.

Figures 2 [Fig F3] [Fig F4] –5 Table 3

##### Type material.

Holotype: CASIZ185253, separated from CASIZ175526, 95% EtOH. Diogo Vaz, São Tomé, Gulf of Guinea, 0°18.89'N, 6°29.39'E, collected by hand/SCUBA, 12–27 m, attached to *Leptogorgia* c.f. *dichotoma*, G. Williams, 29 May 2006. Original label: “S-3”, California Academy of Sciences, San Francisco.

Paratypes: CASIZ173189 (4 specimens) and CASIZ174804, Diogo Vaz, São Tomé, Gulf of Guinea (0°18.89'N, 6°29.39'E), collected by hand/SCUBA, 9–26 m, R. Van Syoc, 29 May 2006; CASIZ 173190 (3 specimens), 174805 (4 specimens), 174806 (2 specimens), and 175526 (7 specimens), Diogo Vaz, São Tomé, Gulf of Guinea (0°18.89'N, 6°29.39'E), collected by hand/SCUBA, 12–27 m, G. Williams, 29 May 2006; CASIZ178655 (2 specimens) and CASIZ178656 (2 specimens), Ilheu BomBom, Príncipe, Gulf of Guinea (1°42'8.8"N, 7°24'14"E), collected by hand/SCUBA, 11 m, R. Van Syoc, 24 Jan 2009.

##### Description.

Exterior of shell with minute bumps, most prominent on parieties. Color variable, white with varying shades of purple concentrated on parietes and basis often at carina side of shell. Radii usually white but can be colored, basis lighter shade of purple to light purplish-red (Fig. 2A–B). Opercular opening round to diamond shaped, small in comparison to shell. Mantle tissue purple near opercular opening. Basis boat shaped (Fig. 2A–B) highly variable depth and length. Basis length of the paratypes 9–21mm. Basis elongated in rostro-carina axis, often deeply indented and/or warped from growing around axis of gorgonian. Carina convex. Rostrum often elongate. Basis and parieties with longitudinal tubes, alae and radii solid. Tubes of basis hollow near wall plate suture where outgrowths from wall plates articulate, otherwise secondarily filled. Wall plates with small, hollow tubes close to external plate surface. Sutural margins denticulated. Shell strong, not disarticulating in sodium hypochlorite solution.

**Figure 3. F3:**
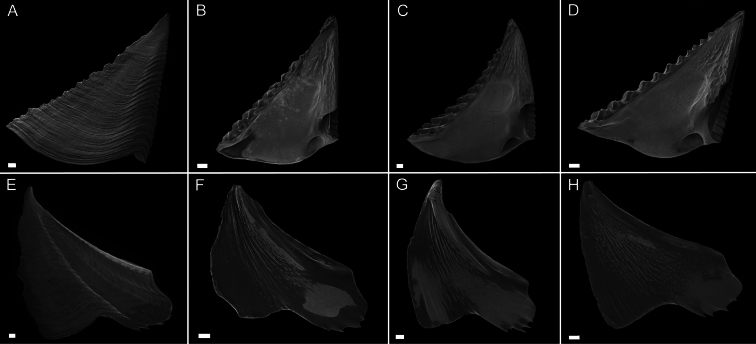
*Conopea saotomensis* sp. n. Opercular plates. **A** scutum exterior (CASIZ175526) **B** scutum interior (CASIZ178655) **C** scutum interior (CASIZ175526) **D** scutum interior (CASIZ174804) **E** tergum exterior (CASIZ175526) **F** tergum interior (CASIZ178655) **G** tergum interior (CASIZ175526) **H** tergum interior (CASIZ174804). Scale bar = 200μm.

Scutum (Fig. 3A–D) with fairly straight tergal and occludent margins, occludent margin may be concave. Basal margin curved. Apex acute. Articular ridge about ⅔ length of tergal margin. Articular furrow present. Adductor ridge absent. Depressor muscle pit deep, medium to large in diameter. Adductor muscle pit shallow. Interior surface of articular ridge and above adductor muscle pit rough with small flat ridges, remainder of interior surface smooth. Interior and exterior of tergum white with varying degrees of purple coloration, most often dark purple, concentrated at apex.

Tergum (Fig. 3E–H) with concave scutal and convex carinal margins, basal margin slightly convex or straight. Apex acute. Basiscutal angle shallow upper corner recessed. Spur smooth, broad, corners rounded approximately ½ to ⅓ width of tergum. Spur margin bearing 3–5 small teeth. Length of spur teeth variable. Spur furrow open. Articular ridge low ⅓ to ½ length of scutal margin. Articular furrow shallow. Depressor muscle crests faint. Interior surface rough with multiple low longitudinal ridges. Coloration matches that of scutum.

**Figure 4. F4:**
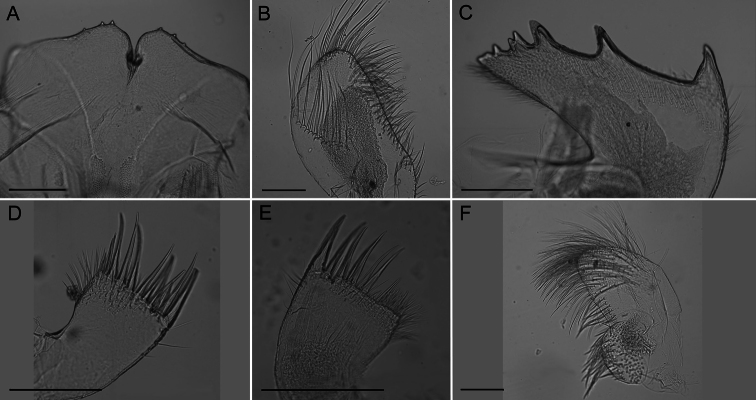
*Conopea saotomensis* sp. n. Mouth parts. **A** labrum (CASIZ174805) **B** mandibular palp (CASIZ174805) **C** mandible (CASIZ174805) **D** maxilla I (CASIZ173190) **E** maxilla I (CASIZ175526) **F** maxilla II (CASIZ178655). Scale bar = 200μm.

Labrum (Fig. 4A) with deep medial notch, 0–3 teeth on both or one side of notch.

Mandibular palp (Fig. 4B) elongate; superior margin convex, partially covered with long setae; apex with long setae; inferior margin with many shorter setae (Fig. 3).

Mandible (Fig. 4C) with 4–5 teeth excluding inferior angle, decreasing in size, tooth 1 largest, well separated from tooth 2, 2 separated from 3 by smaller distance, teeth 3–5 smallest and closest together, teeth 2–5 may be bidentate. Inferior margin densely setose near angle, superior margin and cutting margin below teeth sparsely setose.

Maxilla I (Fig. 4D–E) with 7–10 large thick spines, either evenly distributed or concentrated on ⅔ of the cutting margin near superior margin, remaining cutting margin covered in short fine setae. Many fine short setae along inferior margin near cutting margin and a few fine setae along superior margin near cutting margin.

Maxilla II (Fig. 4F) small, oblong, bi-lobed, covered in long fine setae.

**Figure 5.**
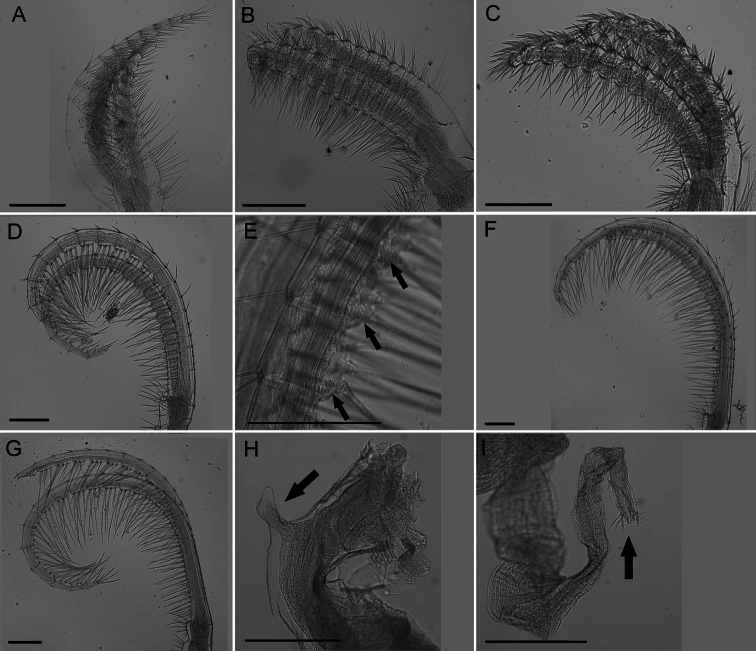
*Conopea saotomensis* sp. n. Cirri and penis. **A** CI (CASIZ174805) **B** CII (CASIZ174805) **C** CIII (CASIZ174805) **D** CIV (CASIZ174805) **E** CIV spines (CASIZ175526) **F** CV (CASIZ174805) **G** CVI (CASIZ174805) **H** penis basidorsal point (CASIZ175526) **I** penis tip (CASIZ175526). Scale bar = 200 μm.

Cirrus I (Fig. 5A) with tapered rami, unequal in length, anterior ramus usually ⅓ longer, posterior ramus more distinct segmentation, setae fine, simple, moderately dense.

Cirrus II (Fig. 5B) rami thick, not tapered, unequal in length but less so than CI, anterior ramus longer, setae simple and dense, segmentation distinct, annulated.

Cirrus III (Fig. 5C) rami thick, slightly tapered, disparate in length, anterior ramus longer, setae simple, dense, thicker than CI or CII, segmentation distinct, annulated.

Cirrus IV (Fig. 5D) rami long, tapered, similar length, segments 1–20, end segment varies, with small spines at base of inferior setae, setae simple, superior setae short, sparse, inferior setae long, dense (Fig. 5E).

Cirrus V (Fig. 5F) rami long, tapered, similar length, setae simple, superior setae short, sparse, inferior setae long, dense

Cirrus VI (Fig. 5G) rami long, tapered, similar length, setae simple, superior setae short, sparse, inferior setae long, dense.

All cirral setae simple.

Penis long, covered in sparse fine setae, large basidorsal point (Fig. 5H), tuft of setae distally (Fig. 5I).

##### Etymology.

*Conopea saotomensis* sp. n.is named after the island from which it was first collected, São Tomé.

##### Distribution.

*Conopea saotomensis* sp. n.is known from São Tomé and Príncipe at depths ranging from 5–34 m living on species of *Leptogorgia* and *Eunicella*.

##### Remarks.

*Conopea saotomensis* sp. n. differs from *Conopea calceola* by the following: distance between scutal depressor muscle pit and articular furrow is wider in *Conopea saotomensis* sp. n. than in *Conopea calceola*; angle between tergal spur and basal margin is smaller in *Conopea saotomensis* sp. n. than in *Conopea calceola*; in *Conopea saotomensis* sp. n. large spines on cutting edge of maxilla span ⅔ or entire length, span entire length in *Conopea calceola*.

**Table 3.** Cirral formula for *Conopea saotomensis* sp. n. (CASIZ 175526; 174805; 178655)

Cirrus	I	II	III	IV	V	VI
Anterior ramus	15–18	13–16	11–13	17–25	27	21–27
Posterior ramus	10–17	11–13	9–12	21–25	22–28	24–29

#### Conopea fidelis sp. n.

urn:lsid:zoobank.org:act:252522DE-A3D4-4FBA-8EF4-5D4014FED8CD

http://species-id.net/wiki/Conopea_fidelis

**Figure 6.**
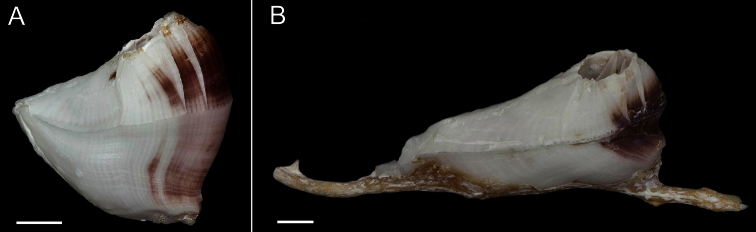
*Conopea fidelis* sp. n. **A** whole shell(CASIZ174322A) **B** whole shell attached to gorgonian axis (CASIZ174322B). Scale bar = 2 mm.

Figures 6 [Fig F7] [Fig F8] –9 Table 4

##### Type material.

Holotype: CASIZ185252, separated from CASIZ174322, 95% EtOH. Ponta Baleia, São Tomé, Gulf of Guinea, 0°2.13'N, 6°33.51'E, collected by hand/SCUBA, 24 m, attached to *Muriceopsis tuberculata*, R. Van Syoc, 30 May 2006. Original label: “RVS – 539, S-6, 30 May 2006, Sao Tome” [handwritten label], California Academy of Sciences, San Francisco.

Paratypes: CASIZ174803 (2 specimens), Diogo Vaz, São Tomé, Gulf of Guinea (0°18.89'N, 6°29.39'E), collected by hand/SCUBA, 9–26 m, R. Van Syoc, 29 May 2006; CASIZ174322 (14 specimens), Ponta Baleia, São Tomé, Gulf of Guinea (0°2.13'N, 6°33.51'E), collected by hand/SCUBA, 24 m, R.Van Syoc, 30 May 2006; CASIZ178651 (2 specimens), Pedra da Gale, Príncipe, Gulf of Guinea (1°43'30.1"N, 7°22'51.5"E), collected by hand/SCUBA, 30 m, R.Van Syoc, 20 Jan 2009.

##### Description.

Exterior of shell covered in very small bumps; color variable, white with pink or light purple on parietes and basis, radii usually white or lighter in color, rostrum often white (Fig. 6A–B). Opercular opening round to diamond shaped, small compared to shell size. Mantle tissue purple near opercular opening. Basis boat shaped (Fig. 6A–B), highly variable depth and length. Basis length of paratypes 14–32 mm. Basis elongated in rostro-carina axis, often deeply indented and/or warped from growing around axis of gorgonian. Carina convex. Rostrum often elongate. Basis with radiating longitudinal tubes, secondarily filled, hollow near wall plate suture. Wall plates with small longitudinal tubes near external surface of shell. Alae and radii lacking tubes. Sutural margins denticulated. Shell wall compartments strongly fused, not disarticulating in sodium hypochlorite solution.

**Figure 7. F7:**
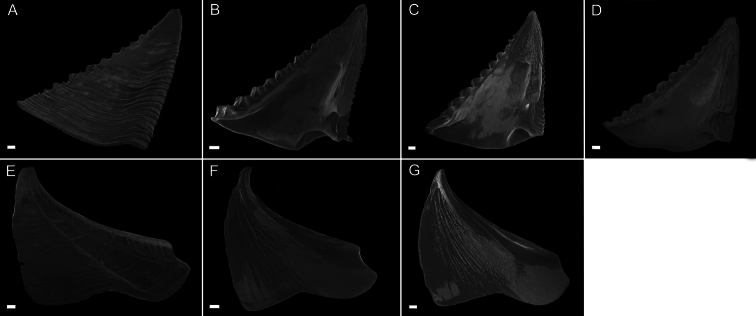
*Conopea fidelis* sp. n.Opercular plates. **A** scutum exterior (CASIZ174803) **B** scutum interior (CASIZ174322) **C** scutum interior (CASIZ178651) **D** scutum interior (CASIZ174803) **E** tergum exterior (CASIZ174803) **F** tergum interior (CASIZ174322) **G** tergum interior (CASIZ178651). Scale bar = 200μm.

Scutum (Fig. 7A–D) with straight to mildly convex tergal margin, occludent margin usually straight , occasionally with curve above basioccludent angle. Basal margin variable, sinuous. Apex subacute. Articular ridge prominent, extending ⅔– ¾ length of scutum. Articular furrow present. Adductor ridge absent. Adductor muscle pit shallow, fairly large. Depressor muscle pit large, deep, broad, may converge with basil margin. Majority of interior surface of scutum smooth, articular ridge and apex with low, flat ridges. Interior and exterior white with varying shades of purple coloration concentrated at apex.

Tergum (Fig. 7E–G) scutal and carinal margins curved. Basal margin straight or slightly curved. Apex acute. Basicutal angle shallow, upper corner recessed. Spur broad, bears no teeth, about ½ to ⅓ width of tergum, spur furrow open. Articular ridge ⅓ to ½ length of tergum. Articular furrow shallow. Depressor muscle crests faint. Interior rough with multiple small ridges. Coloration matches that of scutum.

**Figure 8. F8:**
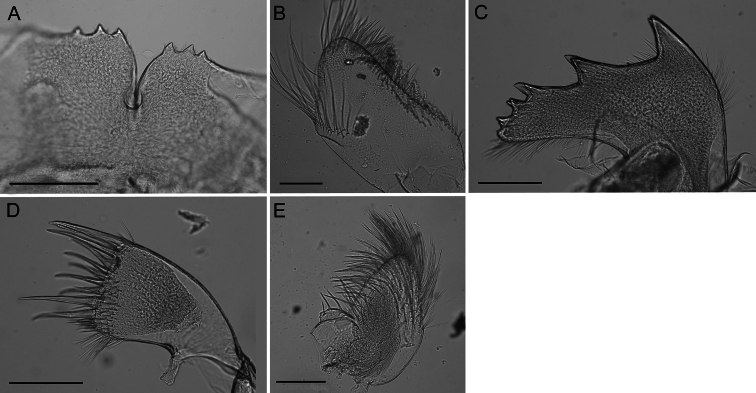
*Conopea fidelis* sp. n. Mouth parts. **A**: labrum (CASIZ174322) **B** mandibular palp (CASIZ174803) **C** mandible (CASIZ174322) **D** maxilla I (CASIZ174322) **E** maxilla II (CASIZ174322). Scale bar = 200μm.

Labrum (Fig. 8A) with deep notch, 0–3 teeth on both or one side of notch.

Mandibular palp (Fig. 8B) slightly convex oval shape, superior margin with curved ridge and sparse fine long setae, inferior margin with dense shorter setae.

Mandible (Fig. 8C) with 4–6 teeth excluding inferior angle, decreasing in size, tooth 1 largest, well separated from tooth 2, 2 separated from 3 by smaller distance, teeth 3-6 smallest and closest together, teeth 1and 2 may be bidentate, 4 and 5 may be bifurcated. Inferior margin densely setose near angle, superior margin and cutting margin below teeth sparsely setose.

Maxilla I (Fig. 8D) with 10–12 large thick spines, many smaller, thinner spines along cutting margin, short setae below margin, dense setae on anterior margin, posterior margin sparsely setose, may have shallow notch.

Maxilla II (Fig. 8E) small, oval shaped, bi-lobed, covered in long, fine setae.

**Figure 9.**
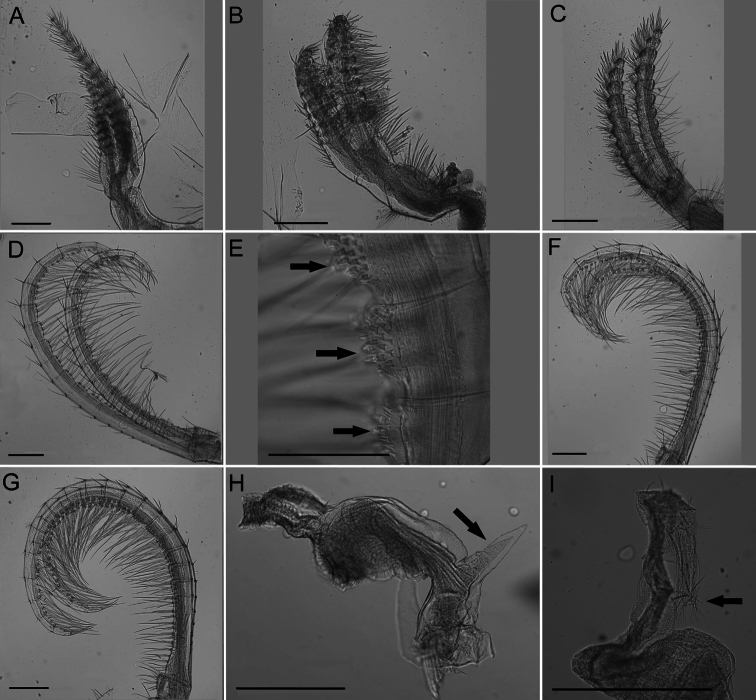
*Conopea fidelis* sp. n. Cirri and penis. **A** CI (CASIZ178651) **B** CII (CASIZ178651) **C** CIII (CASIZ178651) **D** CIV (CASIZ178651) **E** CIV spines (CASIZ178651) **F** CV (CASIZ178651) **G** VI (CASIZ178651) **H** penis basidorsal point (CASIZ174803) **I** penis tip (CASIZ174322). Scale bar = 200μm.

Cirrus I (Fig. 9A) rami densely setose, tapered and unequal in length, anterior rami about ⅓ longer, posterior rami with more annulated segmentation.

Cirrus II (Fig. 9B) rami slightly unequal in length, width thick, segmentation distinct, annulated, thick dense setae.

Cirrus III (Fig. 9C) rami unequal in length but less so than CI, anterior ramus longer, width thick, dense thick setae, segmentation distinct, annulated.

Cirrus IV (Fig. 9D) rami long, tapered, anterior side with long dense setae and small spines at base of setae (Fig. 9E) extending from first to twentieth segment (end segment variable), posterior side with short sparse setae at segment divisions.

Cirrus V (Fig. 9F) rami long with long dense setae on anterior side and short sparse setae at segment divisions on posterior side, about equal in length.

Cirrus VI (Fig. 9G) rami long with long dense setae on anterior side, short sparse setae only at segment divisions of posterior side, similar length.

All cirral setae simple.

Penis long with large basidorsal point (Fig. 9H), covered in short very sparse setae, tuft of setae distally (Fig. 9I).

##### Etymology.

*Conopea fidelis* sp. n. is named so because it is found to be faithful to one host species of gorgonian, *Muriceopsis tuberculata*. From the Latin fidelis: faithful or true.

##### Distribution.

*Conopea fidelis* sp. n. is known from São Tomé and Príncipe at depths ranging from 5–34 m and is found living on the gorgonian *Muriceopsis tuberculata*.

##### Remarks. 

Morphological differences between *Conopea fidelis* sp. n. and *Conopea calceola*are as follows: *Conopea fidelis* sp. n. does not have tergal spur teeth, *Conopea calceola* does; scutal depressor muscle pit may converge with basal margin in *Conopea fidelis* sp. n., it does not in *Conopea calceola*; *Conopea fidelis* sp. n. maximum basis length is longer than that of *Conopea calceola*.

Morphological differences between *Conopea saotomensis* sp. n. and *Conopea fidelis* sp. n.are as follows: *Conopea saotomensis* sp. n. shell color ranges from dark purple to light purplish-red, *Conopea fidelis* sp. n. shell color ranges from light purple to pink; *Conopea fidelis* sp. n . basis length maximum is longer than that of *Conopea saotomensis* sp. n.; length of scutal articular furrow in *Conopea saotomensis* sp. n. is shorter than *Conopea fidelis* sp. n.; scutal depressor pit may converge with basal margin in *Conopea fidelis* sp. n. but not in *Conopea saotomensis* sp. n.; angle between tergal spur and basal margin is smaller in *Conopea saotomensis* sp. n. than *Conopea fidelis* sp. n.; tergal spur teeth present in *Conopea saotomensis* sp. n., absent in *Conopea fidelis* sp. n.; *Conopea saotomensis* sp. n. length of tergal articular ridge is equal or longer to that of *Conopea fidelis* sp. n.; cutting edge spines of maxilla I span entire margin or just ¾ in *Conopea saotomensis* sp. n, span entire margin in *Conopea fidelis* sp. n.; *Conopea fidelis* sp. n. maxilla I may have a notch, *Conopea saotomensis* sp. n. does not.

**Table 4.** Cirral formula for *Conopea fidelis* sp. n.(CASIZ 178651; 174803A; 174322F)

Cirrus	I	II	III	IV	V	VI
Anterior ramus	14–17	11–12	11–12	19–21	23–24	23–25
Posterior ramus	9–11	9–10	10–11	21–22	24–26	23–26

## Discussion

COI has been shown to be useful for delimiting species within the Crustacea (Lefébure et al. 2006) and, in particular, within the Cirripedia (Van Syoc 1995, Wares 2001, Rawson et al. 2003). Costa et al. (2007) found within genus COI divergence levels of crustaceans to range from 4.92% to 31.39%. Van Syoc (1994) found COI divergence levels averaging 1.2% among distantly separated sub-populations of *Pollicipes elegans* (Lesson 1831) (Crustacea: Cirripedia: Scalpelliformes). Van Syoc (1995) and Van Syoc et al. (2010) also found a range of 15%–28% among species of *Pollicipes elegans*, *Pollicipes polymerus* (Sowerby 1833), and *Pollicipespollicipes* (Gmelin 1790). Regarding barnacles, histone genes have been shown to be highly conserved and can be used for deep metazoan phylogenies (Pérez-Losada et al. 2004, 2008, Van Syoc et al. 2010) and for phylogenetic analysis of arthropods (Colgan et al. 1998) and thoracic barnacles (Pérez-Losada et al. 2004). Expected divergence levels of H3 among closely related cirriped species is not known but Van Syoc et al. (2010) found low levels, 0–1.3%, of sequence divergence between species of *Pollicipes*. The overall difference in divergence between genes, higher for mitochondrial COI and lower for nuclear H3, is expected as nuclear genes typically evolve slower than mitochondrial genes for arthropods (Avise et al. 1994, Burton and Lee 1994).COI and H3 divergence levels found for *Conopea saotomensis* and *Conopea fidelis* are both satisfactory for determining a species within Cirripedia.

The barnacles collected from the Gulf of Guinea for this study were originally identified as *C*. cf. *calceola*. The initial identifications were tentative because *Conopea calceola* is not well studied, has a reportedly large distribution, the original species description (Ellis 1758) contains sparse morphological data, and Darwin’s description of *Conopea calceola* (used as our reference for morphology) relied on locality for identification. Ellis (1758) designated the type locality of *Conopea calceola* as the Strait of Gibraltar, which connects the eastern Atlantic Ocean to the Mediterranean Sea. Darwin (1854) recorded the presence of *Conopea calceola* off the west coast of Africa. For his identification he noted that the original description of *Conopea calceola*, by Ellis, does not adequately distinguish any morphological characters for positive identification so he relied on locality, the eastern Atlantic. The *Conopea calceola* specimens from Portugal used in our phylogenetic and morphological analyses match the morphology of the specimens from the coast of Africa that Darwin described as *Conopea calceola*.

Attempts to obtain specimens of *Conopea calceola* from other locations/institutions were unsuccessful. Darwin’s (1854) description of *Conopea calceola* was used as the guideline for the species along with the literature of Hoek (1913), (Hiro 1937), and Ren and Liu (1978). Unfortunately, none of these papers state the number of tergal spur teeth and the images are too poor to count them accurately. Therefore, a comparison of number of tergal spur teeth could not be made. But there is a difference between the 7 Portuguese *Conopea calceola* specimens of this paper and *Conopea saotomensis* sp. n. *Conopea calceola* was found to have 6–9 tergal spur teeth and *Conopea saotomensis* sp. n. 3–5.

### Gorgonian host preference

Barnacles are found permanently attached to many different types of living and non-living substrata. Locating a living substratum, especially one that is mobile or spatially rare, can be challenging for a small marine larva. For example; a gorgonian, a turtle, or a whale is harder to locate than a rock bed. When barnacle larvae locate and settle onto a gorgonian they may be recognizing the substratum, the presence of conspecifics, or both. It has been shown that barnacle larvae can determine where to settle by recognizing pheromone cues from their cohorts (Crisp and Meadows 1962, Knight-Jones 1995, Dreanno et al. 2006, 2007) or chemical cues from their host (Pasternak et al. 2004, Nogata and Matsumura 2005). It has also been shown that gorgonians produce barnacle settlement inducers as well as inhibitors (Standing et al. 1983) and prostaglandins that promote hatching (Clare et al. 1985). The inhibitors are water soluble and so found in the water near the gorgonian whereas the inducers are found absorbed in the gorgonian tissue.

Although the details of the settling barnacle larvae and gorgonian interaction are not completely known, it appears, from our observations (specifically that *Conopea fidelis* sp. n. was found only on *Muriceopsis tuberculata*) that barnacle larvae may be capable of distinguishing between gorgonian species. Of course, more collections, identifications, and laboratory work testing settlement preference would be needed to answer this question.

### Endemism

The possibility that *Conopea saotomensis* sp. n. and *Conopea fidelis* sp. n. are endemic to the Gulf of Guinea Islands is likely for the following reasons: the islands’ distance (approx. 274 km), age (approx. 13 and 30 myo), and historic isolation from mainland Africa; they are not known from any previous locality; many endemic species, terrestrial and marine, are found on the Gulf of Guinea islands (Jones 1994, Measey et al. 2007). However, further sampling from the west coast of Africa is essential to determine if they are indeed endemic.

## Supplementary Material

XML Treatment for Conopea saotomensis

XML Treatment for Conopea fidelis
